# Intercompartmental communication between the cerebrospinal and adjacent spaces during intrathecal infusions in an acute ovine in-vivo model

**DOI:** 10.1186/s12987-021-00300-0

**Published:** 2022-01-04

**Authors:** Anthony Podgoršak, Nina Eva Trimmel, Markus Florian Oertel, Sara Qvarlander, Margarete Arras, Anders Eklund, Miriam Weisskopf, Marianne Schmid Daners

**Affiliations:** 1 Department of Mechanical and Process Engineering, ETH Zurich, Zurich, Switzerland; 2 Division of Surgical Research, University Hospital Zurich, University of Zurich, Zurich, Switzerland; 3 Department of Neurosurgery, University Hospital Zurich, University of Zurich, Zurich, Switzerland; 4 Department of Radiation Sciences, Radiation Physics, Biomedical Engineering, Umea University, Umeå, Sweden

**Keywords:** Cerebrospinal fluid, Bolus infusion, Constant pressure infusion, Hydrocephalus, Intracranial pressure, Intrathecal pressure, Sheep model

## Abstract

**Introduction:**

The treatment of hydrocephalus has been a topic of intense research ever since the first clinically successful use of a valved cerebrospinal fluid shunt 72 years ago. While ample studies elucidating different phenomena impacting this treatment exist, there are still gaps to be filled. Specifically, how intracranial, intrathecal, arterial, and venous pressures react and communicate with each other simultaneously.

**Methods:**

An in-vivo sheep trial (n = 6) was conducted to evaluate and quantify the communication existing within the cranio-spinal, arterial, and venous systems (1 kHz sampling frequency). Standardized intrathecal infusion testing was performed using an automated infusion apparatus, including bolus and constant pressure infusions. Bolus infusions entailed six lumbar intrathecal infusions of 2 mL Ringer’s solution. Constant pressure infusions were comprised of six regulated pressure steps of 3.75 mmHg for periods of 7 min each. Mean pressure reactions, pulse amplitude reactions, and outflow resistance were calculated.

**Results:**

All sheep showed intracranial pressure reactions to acute increases of intrathecal pressure, with four of six sheep showing clear cranio-spinal communication. During bolus infusions, the increases of mean pressure for intrathecal, intracranial, arterial, and venous pressure were 16.6 ± 0.9, 15.4 ± 0.8, 3.9 ± 0.8, and 0.1 ± 0.2 mmHg with corresponding pulse amplitude increases of 2.4 ± 0.3, 1.3 ± 0.3, 1.3 ± 0.3, and 0.2 ± 0.1 mmHg, respectively. During constant pressure infusions, mean increases from baseline were 14.6 ± 3.8, 15.5 ± 4.2, 4.2 ± 8.2, and 3.2 ± 2.4 mmHg with the corresponding pulse amplitude increases of 2.5 ± 3.6, 2.5 ± 3.0, 7.7 ± 4.3, and 0.7 ± 2.0 mmHg for intrathecal, intracranial, arterial, and venous pulse amplitude, respectively. Outflow resistances were calculated as 51.6 ± 7.8 and 77.8 ± 14.5 mmHg/mL/min for the bolus and constant pressure infusion methods, respectively—showing deviations between the two estimation methods.

**Conclusions:**

Standardized infusion tests with multi-compartmental pressure recordings in sheep have helped capture distinct reactions between the intrathecal, intracranial, arterial, and venous systems. Volumetric pressure changes in the intrathecal space have been shown to propagate to the intraventricular and arterial systems in our sample, and to the venous side in individual cases. These results represent an important step into achieving a more complete quantitative understanding of how an acute rise in intrathecal pressure can propagate and influence other systems.

## Introduction

Hydrocephalus is a neurodegenerative disease characterized by disturbed cerebrospinal fluid (CSF) dynamics [1]. Its treatment has been a topic of debate ever since the first documented use of a valved CSF shunt 72 years ago [2]. The ability to design more sophisticated treatment options for CSF-related malfunction is truly limited until gaps in quantitative understanding of CSF dynamics and their communication to the adjacent compartments are filled. Quantifying the interactions between intracranial pressure (ICP), intrathecal pressure (ITP), arterial blood pressure (ABP), and central venous pressure (CVP) will help lay the fundament for a more complete understanding of this communication.

While gaps do exist, there have been ample studies that have helped to form our current methodology in the understanding of CSF dynamics. Infusion tests are well described procedures that have wide-reaching implications in both clinical routine and in research. Clinically, infusion tests are used to predict whether or not normal pressure hydrocephalus patients will respond well to shunt treatment [3]. In research settings, infusion tests are used to induce volumetric pressure changes in the CSF space and evaluate different characteristics, such as pressure volume indices (PVIs) and outflow resistance (R_out_) [4]. Two examples of such infusions are the intrathecal bolus and constant pressure infusions (CPI), used in both clinical and research settings [5–11]. On the one hand, the intrathecal bolus infusion method is a volume-controlled test comprised of a fast infusion of a predefined volume directly into the intrathecal sac. Then, the ITP response to that infusion is measured, including the peak immediately following and the spontaneous relaxation back to baseline. CPI, on the other hand, are continuous infusions that are pressure controlled. Introduced clinically in 1977, ITP is controlled at predefined pressure steps, with the periodic infusion of volume required to control and alter pressure between steps [5].

Previous studies have been invaluable tools to not only add to our understanding of CSF dynamics, but also how the CSF, arterial, and venous sides communicate with each other. It is postulated that CSF has its origin in the choroid plexus, the ependyma, and the parenchyma [12–14]. Its dynamics are driven by the cardiac [15] and respiratory [16, 17] cycles, as well as bulk flow from the point of production to the point of absorption [18, 19]. However, a single study that quantifies the propagation of pressure between the CSF, arterial, and venous spaces in a single in-vivo model is yet to be completed.

In this in-vivo ovine study, the CSF, arterial, and venous pressures are measured simultaneously during intrathecal bolus and constant pressure infusions. ICP, ITP, ABP, CVP, and the relationships among them are presented and quantified to provide insights into the physiologic reactions under volume-induced CSF pressure changes, including mean pressure reactions as well as changes to individual pulse pressures.

## Methods

### Ethical statement

Animal housing and all experimental procedures were approved by the local Committee for Experimental Animal Research (Cantonal Veterinary Office Zurich, Switzerland) under the license number ZH119/2019, and were conforming to the European Directive 2010/63/EU of the European Parliament and the Council on the Protection of Animals used for Scientific Purposes, as well as to the Guide for the Care and Use of Laboratory Animals [20].

### Anesthesia and animal instrumentalization

Anesthesia was induced by i.v. injection of ketamine hydrochloride [Ketasol^®^-100 ad us.vet.; Dr. E. Graeub AG, Berne, Switzerland; 3 mg/kg body weight (BW)] in combination with midazolam (Dormicum^®^, Roche Pharma (Schweiz) AG, Reinach, Switzerland; 0.2 mg/kg BW) and propofol (Propofol^®^- Lipuro 1%, B. Braun Medical AG; Sempach, Switzerland 2–4 mg/kg/h; 2–5 mg/kg BW). After intubation, anesthesia was maintained by positive pressure ventilation (fresh gas flow 1–1.5 L/min, 12–15 breaths/min, tidal volume 10–15 mL/kg, FiO2 0.5) of 2–3% isoflurane in oxygen/air mixture and a continuous infusion pump applying propofol. Throughout the procedure the animals additionally received a continuous intravenous infusion of sufentanil (Sufenta^®^ Forte, Janssen-Cilag AG, Zug, Switzerland; 0.05 mg/kg/h).

**Fig. 1:**
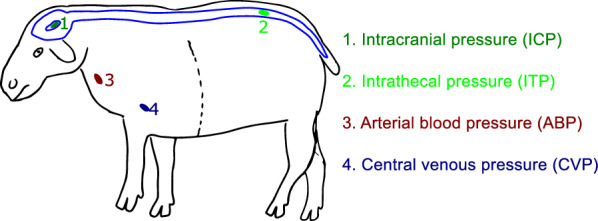
Pressure measurement locations considered in this study. 1, Intracranial pressure (ICP); 2, Intrathecal pressure (ITP); 3, Carotid Arterial Blood arterial blood pressure (ABP); 4, Central Venous venous pressure (CVP)

In all sheep (Table 1), percutaneous ultrasound guided placement of a carotid arterial line (4 Fr) and a multilumen jugular vein catheter (AeroGuard Blue, Arrow^®^, Teleflex Medical Europe Ltd., Ireland) was performed (Fig. 1). For measurement of ICP, a 9 Fr catheter (Ref. 55-3000, Neuromedex GmbH, Hamburg, Germany) was placed through a right frontal burr hole trephination approximately 2 cm from the sagittal suture. This catheter was placed in the right lateral ventricle, confirmed via inspection of CSF egression along the catheter, and anchored with Ethicon Bonewax (Johnson & Johnson Medical Ltd., Livingston, UK) to avoid CSF leakage. A 4.5 Fr Neuromedex catheter (Ref. 61-1400) was placed in the intrathecal sac to measure ITP via a laminotomy at level L6-7. The same access was used for the spinal needle (Perifix 310 mini set, 5 Fr, B.Braun Melsungen AG, Melsungen, Germany) placement to perform the infusion experiments. In both cases, the catheter and needle were anchored with Ethicon Bonewax (Johnson and Johnson Medical Ltd.) to avoid CSF leakage. Hydrostatic equivalence was maintained between the intraventricular and intrathecal transducers by zeroing them to atmospheric pressure at level of the lateral ventricles and the arterial and venous sensors at the right atrial level. All transducers were fixated to the skin with either surgical clamps or sutures. After instrumentalization the sheep was placed in sternal position throughout the experiment, mimicking horizontal position in humans at which the ITP and ICP are assumed to not be influenced by hydrostatic variations.

**Table 1:** Demographic data of all sheep used in this study

Ewe demographic data		
Identifier	Age	Weight (kg)
A	2 years 0 months	93.5
B	2 years 8 months	85.0
C	3 years 0 months	72.5
D	4 years 0 months	67.5
E	2 years 1 month	75.5
F	5 years 0 months	60.0

### Experimental protocol

The goal-driven experimental protocol was designed to adequately illuminate how ICP, ITP, ABP, and CVP propagate with each other across a range of pre-defined and well-controlled scenarios. Part of this protocol was a detailed infusion study, which contained intrathecal bolus and CPI. To ensure accurate control, an automated infusion apparatus was used [21]. The apparatus allowed for volume and pressure control of the bolus and CPI, respectively. The peristaltic pump of the pressure controller induced additional oscillations while regulating pressure lower than the roller frequency, which were subsequently filtered using a Butterworth forward–backward band stop filter of order 4 with a stopband of 0.2–0.5 Hz. To perform the infusions, the apparatus was connected to the needle placed in the lumbar thecal sac and was verified to be at the same relative height as the ITP transducer to avoid hydrostatic variations.

Bolus infusions of 2 mL Ringer’s solution were injected directly into the lumbar intrathecal space. Relaxation time was set to 7 min or until baseline values were resumed. CPI contained six unique elevated pressure steps of 3.75 mmHg starting from a sheep-specific pressure that depended on the initial baseline pressure.

### Data acquisition and analyses

All data were acquired using the commercially available software, Ponemah v5.1 (Data Science International, St. Paul, USA) with the ACQ-7700 acquisition unit using the Universal XE and ABCD 4 to amplify the signal. All data were acquired at a sampling frequency of 1 kHz, discriminated to 100 Hz and analyzed using custom scripts written in Python 3.7.10 (Open Source, Python Software Foundation, Willmington, Delaware, United States). Baseline mean pressures and pulse amplitudes were measured as 5-min arithmetic means before the first infusion; these values were then used as the defined baseline values for calculation. Values are reported as mean ± SD. The frequency spectrum of the raw data was analyzed using discrete fast Fourier transform (FFT).

### Mean reactions

To gain initial insights into how the pressures of interest reacted to the volumetric pressure changes induced by the infusions, mean reactions were calculated after the data was pre-processed. Outliers were rejected by using a z-score rejection method with a σ_crit_ of 3. Then, to remove effects of the cardiac and respiratory waveforms, the data was lowpass filtered using a 4th order forward/backward Butterworth filter with a cutoff frequency of 0.1 Hz (10 s periods). This excluded physiologic effects while retaining the bulk effects seen by infusions. Mean changes were calculated for the bolus and CPI. Temporal offsets were calculated using cross-correlation.

**Fig. 2:**
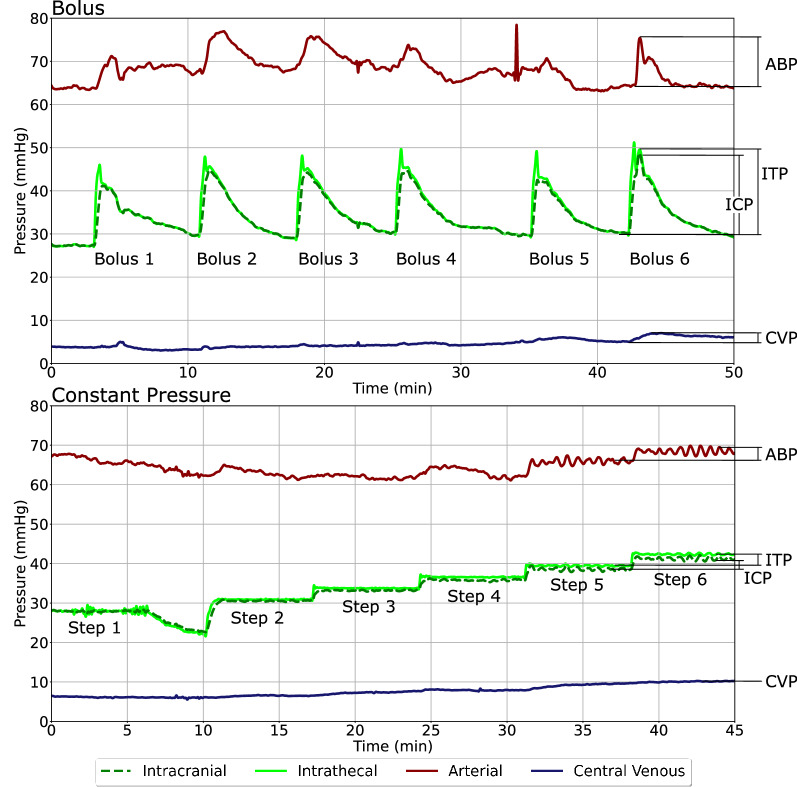
Visualization of how mean changes in pressure were calculated for bolus infusions (top) and constant pressure infusions (bottom). Individual pressure steps used for the constant pressure infusions can be seen in bottom. Horizontal lines depict the upper and lower limits used for calculations. Please note that the timeframe represents elapsed time. Shown on data from Sheep F. ICP intracranial pressure; ITP intrathecal pressure; ABP arterial blood pressure; CVP central venous pressure

The pressure changes over the six discrete bolus infusions are calculated as peak post-infusion pressure minus peak pre-infusion pressure of the lowpass filtered data (Fig. 2). The constant pressure infusions are split into six discrete pressure steps. The mean pressure steps, calculated as the averages over the entire length of the pressure step once the pressure has stabilized, was calculated.

### Pulse amplitude reactions

**Fig. 3:**
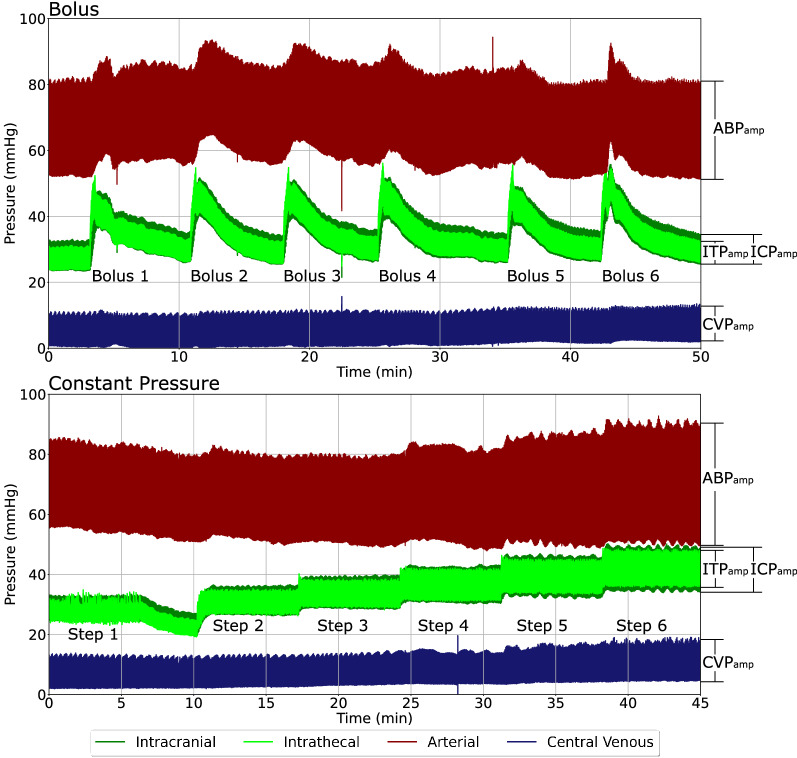
Visualization of how reactions were calculated during bolus infusions (Top) and constant pressure infusions (Bottom). Individual pressure steps used for the constant pressure infusions Horizontal lines illustrate example upper and lower limits of pressure waveforms. All amplitudes are peak-to-peak. Please note that the timeframe represents elapsed time. ICP_amp_, intracranial pressure pulse amplitude; ITP_amp_, intrathecal pressure pulse amplitude; ABP_amp_, arterial blood pressure pulse amplitude; CVP_amp_, central venous pressure pulse amplitude

Pressure signals were evaluated over lumbar intrathecal bolus and constant pressure infusions. All amplitudes were calculated as the difference between the systolic and diastolic pressures (Fig. 3). Pulse amplitudes during bolus infusions were calculated as arithmetic averages 10 s pre and post infusion. Amplitudes during constant pressure infusions were calculated as arithmetic averages over the length of the entire pressure step.

### CSF outflow resistance

**Fig. 4:**
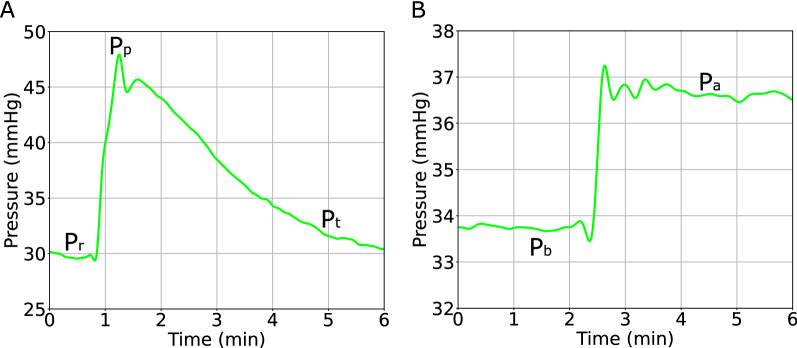
**A** Bolus infusion method for calculating R_out_. **B** CPI method for calculating R_out_. Pressure curves taken from sheep F. Please note that the timeframe represents elapsed time. Pr, resting pressure pre-infusion; Pp, peak pressure post infusion; Pt, relaxation pressure at time t post infusion; Pb, pre-infusion baseline pressure; Pa, average pressure during infusions

CSF outflow resistance (R_out_) was calculated using the methods outlined in Eklund et al. [10] using ICP data during bolus (Eqs. 1, 2) and constant pressure infusion (Eq. 3) methods. For the bolus method (CSF outflow resistance (R_out_) was calculated using the methods outlined in Eklund et al. [10] using ICP data during bolus (Eqs. 1, 2) and CPI (Eq. 3) methods. For the bolus method (Fig. 4A), the pressure–volume index (PVI) is first calculated with:

1 $$PVI = { }\frac{\Delta V}{{\log \left( {\frac{{P_{p} - P_{0} }}{{P_{r} - P_{0} }}} \right)}}{ ,}$$

where $\Delta {\text{V}}$ is the amount of infused volume, ${\text{P}}_{{\text{p}}}$ is the peak pressure post-infusion, ${\text{P}}_{{\text{r}}}$ is the resting pressure just before the infusion, and P_0_ is the reference pressure (assumed to be zero). Then, R_out_ can be determined from the spontaneous relaxation curve post-infusion (Eq. 2),

2 $$R_{out} = \frac{{tP_{r} }}{{PVI*\log \left[ {\frac{{\left( {\frac{{P_{t} }}{{P_{p} }}} \right)\left( {P_{p} - P_{r} } \right)}}{{P_{t} - P_{r} }}} \right]}} ,$$

where ${\text{t}}$ is the time post-infusion and ${\text{P}}_{{\text{t}}}$ is the relaxation pressure at time ${\text{t}}$. Relaxation pressures were taken at 1-, 2-, 3-, and 4-min post bolus infusion to calculate R_out_ as an average across these four timepoints.

For the CPI method (Fig. 4B), infusion pressures were averaged across the entire pressure step once pressures attained steady state. Each infusion pressure step is taken as the pre-infusion baseline pressure (P_b_) and average pressure during (P_a_) infusion and divided by the infusion rate required to maintain the pressure at the next step (Eq. 3).

3 $$R_{out} = \frac{{P_{a} - P_{b} }}{{Q_{inf} }}$$

## Results

### Baseline (pre-infusions)

**Fig. 5:**
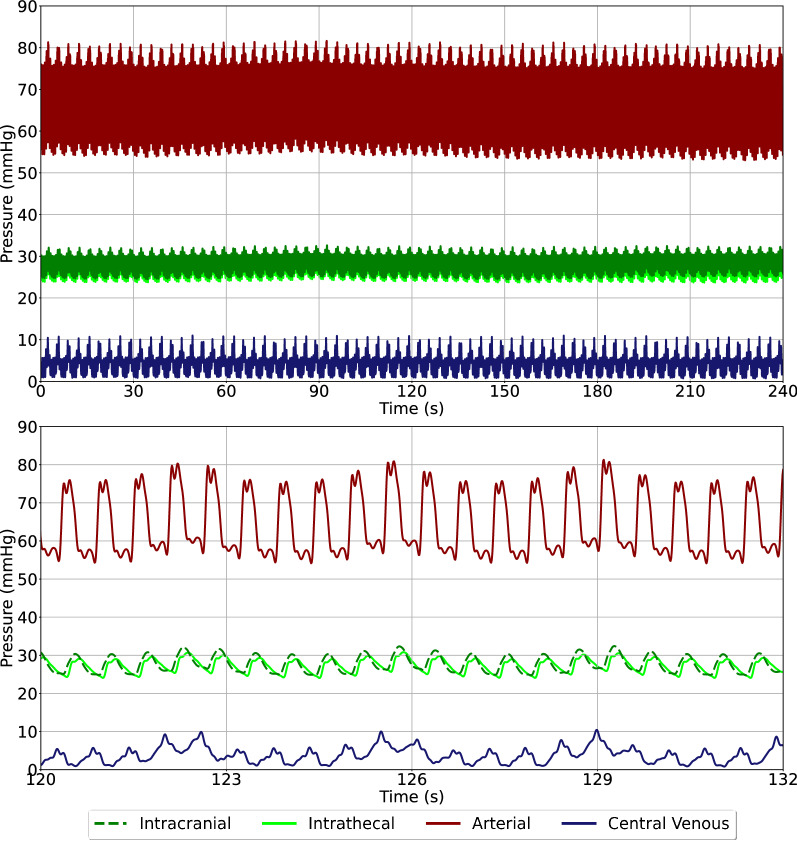
Top: representative baseline waveforms observed pre-infusion. Please note that the timeframe represents elapsed time. Bottom: close up view of a 12 s interval showing the respective pressure pulses of arterial, intrathecal, intracranial, and central venous recordings and additional slow oscillations caused by respiration. Data taken from sheep F

There are two distinct waveforms present in the pre-infusion baseline data, corresponding to the cardiac and respiratory cycles. (Fig. 5). In sheep A, B, C, and F, ITP and ICP are directly comparable at baseline whereas sheep D, and E show a disconnect in pressures, even though anatomical communication is assumed (Table 2). Sheep B and F have the largest baseline ITP which also corresponds to the largest baseline ITP_amp_. Arterial and venous pressures were observed to be within expected values. Mean ITP and ICP were observed to be within the expected physiologic range of 0–20 mmHg [22], yet there was a large variation between animals.

**Table 2:** All baseline (pre-infusion) data from all sheep

Sheep	ITP (mmHg)	ITP_amp_ (mmHg)	ICP (mmHg)	ICP_amp_ (mmHg)	ABP (mmHg)	ABP_amp_ (mmHg)	CVP (mmHg)	CVP_amp_ (mmHg)
A	0.4 ± 0.2	0.3 ± 0.0	2.2 ± 0.1	0.4 ± 0.1	84.6 ± 1.5	15.3 ± 0.4	4.6 ± 0.1	4.7 ± 0.2
B	23.9 ± 1.2	3.2 ± 0.1	20.7 ± 0.8	3.2 ± 0.3	91.8 ± 1.0	18.0 ± 0.8	5.3 ± 0.2	3.4 ± 0.0
C	6.2 ± 0.9	1.2 ± 0.0	6.5 ± 0.2	3.2 ± 0.1	65.9 ± 0.7	37.4 ± 0.4	4.7 ± 0.9	2.4 ± 0.0
D	0.0 ± 0.3	1.1 ± 0.0	10.0 ± 0.0	1.4 ± 0.0	83.8 ± 2.8	12.7 ± 0.2	2.9 ± 0.3	0.8 ± 0.0
E	5.6 ± 4.5	1.7 ± 0.1	16.7 ± 0.0	0.4 ± 0.0	89.8 ± 0.4	15.8 ± 0.2	1.3 ± 0.0	4.9 ± 0.1
F	27.2 ± 0.2	5.7 ± 0.1	27.2 ± 0.3	6.9 ± 0.2	64.4 ± 0.7	26.5 ± 0.3	3.9 ± 0.1	4.7 ± 0.1

ITP, intrathecal pressure; ITP_amp_, intrathecal pressure pulse amplitude; ICP, intracranial pressure; ICP_amp_, intracranial pressure pulse amplitude; ABP, arterial blood pressure; ABP_amp_, arterial blood pressure pulse amplitude; CVP, central venous pressure; CVP_amp_, central venous pressure pulse amplitude

### Baseline frequency analysis of raw data

**Fig. 6:**
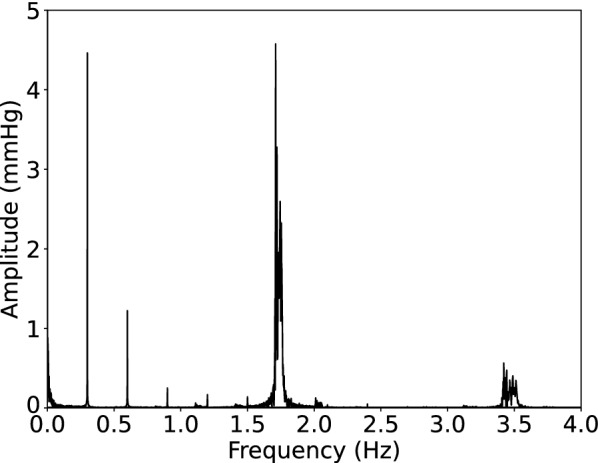
Representative frequencies of sheep F assessed by fast Fourier transform. The fundamental respiratory frequency at 0.30 Hz and the cardiac frequency at 1.74 Hz with their respective harmonics (0.60, 0.90, 1.20, and 1.50 Hz and 3.50 Hz, respectively) are depicted

There were two unique physiologic frequencies observed in all sheep, corresponding to influences from the cardiac and respiratory cycles (Fig. 6). The fastest frequency component stems from the cardiac cycle, with an average heart rate (N = 6) of 1.6 ± 0.6 Hz (98 ± 4 bpm). The second primary waveform comes from the controlled ventilation of the sheep, with an average frequency of 0.3 ± 0.0 Hz (20 breaths per minute).

## Bolus infusions

**Fig. 7:**
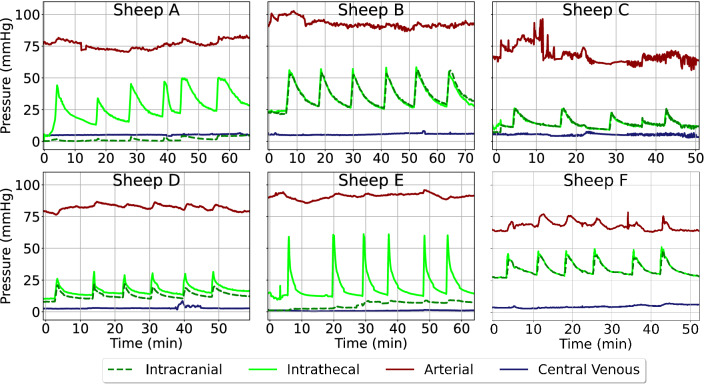
Reactions of ICP, ITP, ABP, and CVP to an intrathecal bolus infusion in all sheep (A–F). Each spike is representative of a bolus infusion. Infusions contained 2 mL of Ringer’s solution, repeated six times per sheep. Please note that the timeframe represents elapsed time and that, in Sheep B and F, ICP and ITP are near equivalent. ICP, intracranial pressure; ITP, intrathecal pressure; ABP, arterial blood pressure; CVP, central venous pressure

Across all six sheep, reactions in intrathecal pressure were observed, with intrathecal to intracranial communication being observed in all sheep to varying degrees and an average peak-to-peak time delay of 0.31 s (Fig. 7). Each sheep had a different reaction to the same infused volume and rate of infusion according to their body size (Table 1), where a larger sheep led to a more muted reaction to the infusion. Sheep E had the most pronounced reaction in ITP—with minimal communication to the intracranial space.

ABP reactions were observed in all sheep. Interestingly, even when there was minimal effect on the ICP from the induced increase in ITP, as in sheep A and E, there was still an acute reaction in the ABP. Sheep C experienced considerable extrasystolic formations (35–50 min), which limit conclusions that can be drawn from the final two bolus infusions. There was minimal acute reaction to the infusions in CVP, however a mild increase across the entire length of the infusions is observable.

In sheep B, C, D, and F, cranio-spinal communication existed with an acute rise in mean ITP due to the infusion propagating to the intracranial space with corresponding reactions in the pulse pressure (Table 3), with a regression coefficient of 0.98. In sheep A and E, there was minimal cranio-spinal communication: only a small increase in ICP occurred following the acute increase in ITP. Across all sheep, ABP increased following the bolus to varying extent. In sheep A where there was minimal communication between ITP and ICP, the ABP reaction was larger than in sheep B and C where ITP to ICP communication existed. ABP_amp_ reacted the most in sheep F, where the largest ABP reaction was observed. Across all sheep and all measured pressures, reactions were observed in pulse pressure. Of all pressures, ITP reacted the most to the bolus infusions across all sheep, with an average of 2.4 ± 0.3 mmHg peak-to-peak amplitude increase.

**Table 3:** Reactions of ITP, ITP_amp_, ICP, ICP_amp_, ABP, ABP_amp_, CVP, CVP_amp_, during bolus infusions

Sheep	ΔITP (mmHg)	ΔITP_amp_ (mmHg)	ΔICP (mmHg)	ΔICP_amp_ (mmHg)	ΔABP (mmHg)	ΔABP_amp_ (mmHg)	ΔCVP (mmHg)	ΔCVP_amp_ (mmHg)
A	24.9 ± 2.3	2.5 ± 0.4	1.9 ± 0.3	1.4 ± 0.3	3.2 ± 0.9	1.1 ± 0.3	0.0 ± 0.1	0.3 ± 0.1
B	24.4 ± 0.8	2.7 ± 0.2	24.2 ± 1.0	1.1 ± 0.2	2.3 ± 0.8	1.3 ± 0.7	0.0 ± 0.2	0.1 ± 0.1
C	14.1 ± 2.0	2.1 ± 0.4	13.8 ± 1.1	1.3 ± 0.3	0.8 ± 0.9	1.0 ± 0.4	-0.0 ± 0.1	0.0 ± 0.1
D	14.0 ± 0.5	2.6 ± 0.4	9.8 ± 0.7	1.2 ± 0.2	4.1 ± 0.5	1.4 ± 0.3	0.0 ± 0.1	0.1 ± 0.1
E	42.2 ± 0.5	2.4 ± 0.3	2.3 ± 0.4	1.3 ± 0.2	0.7 ± 0.9	0.7 ± 0.1	0.1 ± 0.1	0.3 ± 0.1
F	13.8 ± 0.5	1.9 ± 0.1	13.5 ± 0.5	1.0 ± 0.2	8.4 ± 0.9	2.4 ± 0.4	0.6 ± 0.2	0.3 ± 0.2

ITP, intrathecal pressure; ITP_amp_, intrathecal pressure pulse amplitude; ICP, intracranial pressure; ICP_amp_, intracranial pressure pulse amplitude; ABP, arterial blood pressure; ABP_amp_, arterial blood pressure pulse amplitude; CVP, central venous pressure, CVP_amp_, central venous pressure pulse amplitude

## Constant pressure infusions

**Fig. 8:**
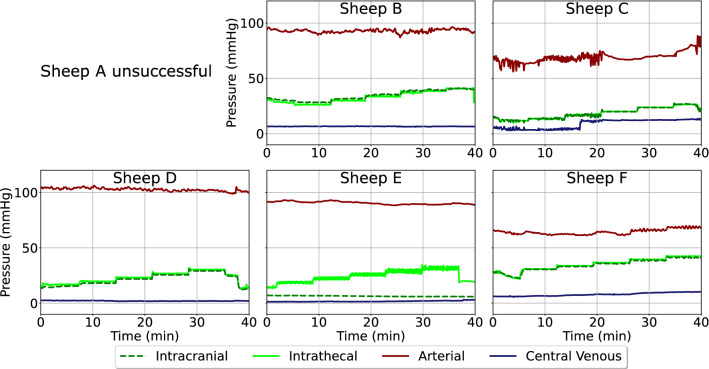
Reactions ICP, ITP, ABP, and CVP to intrathecal constant pressure infusions in all sheep (A–F). Constant pressure infusions were completed by setting a baseline for each sheep and increasing and maintaining the ITP at 3.75 mmHg above the previous step for 7 min. Constant pressure infusions were unable to be completed in sheep A due to hardware limitations. Please note that the timeframe represents elapsed time. ABP, arterial blood pressure; ICP, intracranial pressure; ITP, intrathecal pressure; CVP, central venous pressure

Similar patterns in ITP to ICP communication as during bolus infusions were observed during the CPI (Fig. 8), with a regression coefficient of 0.91 and peak-to-peak offset of 0.34 s. ICP to ITP communication was seen only in those sheep where a reaction was observed during bolus infusions. There was a less obvious connection between ITP and ABP, however reactions were still observed in sheep C and F. Extrasystoles propagated from the arterial to the CSF space in sheep C (minute 0–20). Beyond this, a rise in ITP and ICP was followed by an increase in ABP. In sheep F, the stepwise pressure reaction seen in ITP and ICP was also observed to propagate to the arterial side with a corresponding venous pressure increase.

The constant pressure infusions yielded reactions for those sheep who already showed communication between the two CSF spaces (Table 4). Different to the bolus infusions, a noticeable increase in CVP was observed over the length of the constant pressure infusion study.

**Table 4:** Mean pressures across sheep B, C, D, and F during intrathecal constant pressure infusions

Step	ITP (mmHg)	ITP_amp_ (mmHg)	ICP (mmHg)	ICP_amp_ (mmHg)	ABP (mmHg)	ABP_amp_ (mmHg)	CVP (mmHg)	CVP_amp_ (mmHg)
1	20.4 ± 3.8	7.1 ± 1.5	20.7 ± 4.3	5.9 ± 1.1	81.2 ± 9.8	22.4 ± 1.5	4.7 ± 1.0	7.7 ± 0.8
2	22.9 ± 4.1	8.8 ± 1.2	23.4 ± 4.5	6.2 ± 1.1	81.7 ± 9.8	24 ± 1.3	4.8 ± 1.1	7.7 ± 0.8
3	26.1 ± 4.4	8.6 ± 1.4	26.8 ± 4.5	6.7 ± 1.2	81.9 ± 10.0	23.7 ± 1.5	6.2 ± 1.3	7.8 ± 0.9
4	29.3 ± 4.5	8.3 ± 2.0	29.9 ± 4.3	7.6 ± 1.5	81.7 ± 9.5	24.2 ± 2.4	7.1 ± 2.1	7.9 ± 0.9
5	32.2 ± 4.0	8.9 ± 2.1	32.7 ± 3.8	8.0 ± 1.7	83.0 ± 8.9	26.7 ± 3.1	7.5 ± 2.2	8.3 ± 1.1
6	35.0 ± 3.9	9.6 ± 2.1	36.2 ± 4.2	8.4 ± 1.9	85.4 ± 7.8	30.1 ± 2.8	7.9 ± 2.4	8.4 ± 1.2

ITP_amp_, intrathecal pressure pulse amplitude; ICP_amp_, intracranial pressure pulse amplitude; ABP_amp_, arterial blood pressure pulse amplitude; CVP_amp_, central venous pressure pulse amplitude

All pressure amplitudes measured appear to have a direct relationship with mean ICP. ABP_amp_ had the largest increase over the length of the experiment with a 34.8% increase in pulse amplitude. Interestingly, while mean CVP had little reaction, a 9.1% increase in amplitude was observed.

## Outflow resistance

Table 5 shows the R_out_ across those sheep that showed ITP to ICP communication during bolus and CPI. R_out_ carried average values of 51.6 ± 7.8 and 74.8 ± 4.7 mmHg/mL/min for the bolus and CPI, respectively. Sheep A and E were non-communicating and therefore were not included in this analysis.

**Table 5:** R_out_ across communicating sheep for bolus and constant pressure infusions

Sheep	Bolus	Constant pressure
A	N/A	N/A
B	83.0 ± 9.3	71.6
C	38.4 ± 7.1	79.1
D	39.8 ± 3.5	78.4
E	N/A	N/A
F	45.3 ± 2.5	69.9

## Discussion

### Infusions

Across the entirety of the infusion study, ITP to ICP communication existed under intrathecal infusions in sheep B, C, D, and F. The study further showed that not only does the induced increase in ITP propagate to the cranial CSF space, but also induces a considerable compensatory reaction of the ABP as well as a more muted reaction in CVP. In sheep A and E, the induced increases in ITP only yielded minimal reaction in ICP, indicating a potential obstruction of the fluid communication between the spinal and cranial CSF spaces.

It is postulated that there exists a link between elevations of mean ICP and correspondent increases in pulse amplitude, as directed via the pulsatility curve [11]. In sheep where cranio-spinal communication exists, this is the case; pulse amplitude increases in proportion to the mean pressure due to the reduced compliance, albeit to a much lower extent than would be expected when compared to the equivalent human phenomena [11, 23], indicating that the compliance reserve in sheep may be separated into two discrete components: an ICP dependent on the venous compartment which dominates at lower pressures and appears to already be exhausted at around 20–25 mmHg, and a Dural distensibility component which is constant for all ICP and therefore remains at the higher pressures observed in the bolus infusions. In sheep A and E, where minimal ITP to ICP communication existed during the infusion study, there were still pulse reactions in ICP during bolus infusions. This still agrees with the commonly-held doctrine that changes in pulse amplitude are driven by changes in compliance [24], as there is a decrease in the volume reserve correspondent to a mechanical reduction in compliance, at least in the spinal compartment. This compliance reduction would then lead to higher amplitudes as long as some, even minimal, communication exists.

The bolus infusion, while a viable tool for assessing physiologic and pathologic relationships of the CSF and adjacent spaces, has one considerable limitation: it consistently underestimates CSF outflow resistance using Marmarou’s model [25]. The underlying mechanisms causing these weaknesses have led to disagreement within the field. Bottan et al. [26] argue that the inherent viscoelastic behavior of the brain causes the ICP spontaneous relaxation curve to be shaped such that Marmarou’s equations do not yield accurate results. A mean result of 51.6 ± 7.8 mmHg/mL/min using the bolus method does hold literature agreement (51.0 ± 7.8 mmHg/mL/min) [27], but possible effects of viscoelasticity were not considered and are topics of further investigation. CPI are believed to yield results closer to the true physiology [26], due to the fact that there is a longer exposure period to artificially increased ICP and viscoelasticity does not play as large of a role. Furthermore, the exponential relationship between ICP and volume is only valid at higher pressures, making the Marmarou model potentially ill-suited for this type of analysis [11] as the sheep baseline pressures may be too low. The constant pressure method used yielded an average R_out_ of 74.8 ± 4.7 mmHg/mL/min, which is slightly higher than other values reported in the literature (66.9 ± 14.5 mmHg/mL/min) [27]. However, it is important to note that the literature on sheep R_out_ remains sparse and that the same methods were used with the same potential weaknesses, therefore statements directly comparing cross-study values cannot yet be made.

### Physiology

To understand changes observed in ICP, ITP, ABP, and CVP, both in mean and in pulse, one must first understand the mechanical and physiologic properties that are at play during acute dynamic pressure changes. A volumetric increase in the intrathecal space (due to infusions) induces a corresponding increase both in mean pressure (due to the increase in fluid volume) and pulse pressure (due to reduced compliance). This pressure propagates to the intracranial space, leading to dynamic increases by the same mechanical mechanisms. This acute induction of increased ICP is assumed to cause cerebral perfusion pressure to decrease, making it more difficult for the brain tissue to be properly perfused [28]. Consequently, ABP and pulse amplitude could increase as a compensatory mechanism to maintain cerebral perfusion. While this effect is most easily observed in sheep F, it can also be observed in all sheep where ITP to ICP communication existed. Minimal CVP reaction was observed across the infusion study both in mean and pulse, corresponding to previous literature stating that elevated ICP serves to attenuate venous pulse reactions [29].

Positive pressure ventilation was used to standardize respiration rate and to avoid increased pCO2 due to insufficient spontaneous respiration caused by respiratory depressing drugs (e.g., opioids). It was reported that ICP is not increased by positive pressure ventilation and CVP and arterial blood pressure only increase with positive end-expiratory pressure [30], which was set to 0 in our setting. That together with pCO2 directly altering cerebral perfusion and ICP justifies the mechanical ventilation in this acute model.

In common clinical settings, infusion tests are conducted via lumbar needles inserted into the intrathecal space [31]. One study from Lenfeldt et al. [9] showed that the measurement of intrathecal CSF pressure being used as an analog for ICP is indeed valid, yielding an excellent correlation between lumbar ITP and ICP with a regression coefficient of 0.98. In those sheep in the present study where fluid communication was observed (sheep B, C, D, and F), excellent correlation between ITP and ICP was also observed, with coefficients of 0.91 and 0.98 for bolus and CPI, respectively. These results further encourage that when communication exists between the compartments of the CSF space, ITP can be considered as an analog for ICP. Furthermore, because two sheep did not exhibit any communication, it might be important to confirm communication between the intracranial and intrathecal CSF regions by diagnostic imaging prior to the investigations.

In the study of Malm et al. [32], the normal median R_out_ in healthy humans is 8.6 mmHg/mL/min. Moreover, there is a large variation in R_out_ and not normally distributed. In presumably healthy sheep, this study yielded a R_out_ of 51.6 ± 7.8 and 77.8 ± 14.5 mmHg/mL/min for bolus and CPI, respectively, considerably higher than the equivalent human parameter however also showing large variation between subjects. Moreover, it must be restated that bolus infusions consistently underestimate R_out_, [26] which leads to different results for a single clinical parameter. However, there are further anatomic and physiologic considerations that must be made. Primarily, the anatomic dimensions of the ovine CSF space are much smaller than the corresponding human CSF space, being estimated to contain only 25 mL of CSF compared to mL in humans [27, 33]. This, paired with the diameter of the CSF pathways being smaller, leads to naturally higher resistance. The second consideration that must be made is in the CSF formation rate of sheep, estimated to be only 7.8 ± 0.7 mL/h while the comparable human parameter is in excess of 24 mL/h [5, 27]. These considerations synergistically combined potentially limit the direct comparability of these two species’ R_out_.

### Clinical implications

While the primary motivation of this study focused on further understanding the contribution of adjacent spaces to CSF dynamics, our work also provides key clinical implications for the treatment of CSF-related disorders. Knowledge about hydrocephalus etiologies, while being profoundly researched, still contains gaps e.g., about the potential influence of ICP, ITP, ABP and CVP variations on its pathophysiology, that could potentially limit therapeutic innovation. Our results suggest a strong communication between ABP and cranio-spinal CSF pressure, reinforcing previous findings [22] and quantifying an important physiologic connection. Finally, the establishment of the sheep as a model to investigate CSF dynamics can help in the development a mechatronic platform for the further development and innovation of shunts [34].

### Limitations

This study was conducted with six sheep, which might limit the applicability of the results and does not allow for definitive conclusions. Use of mechanical positive pressure ventilation has not only could have an impact on venous and thoracic pressure but also on bulk flow of CSF, which may have influenced our results. Furthermore, as sheep are quadrupeds, there are elements specific to their physiologic fluid dynamics that limit their transferability to humans. Abdominal pressures were not considered in the present study but will be investigated in a future study. While the consideration of ICP, ITP, ABP, and CVP in CSF dynamics research is a promising approach, determination of abdominal pressure behavior is highly relevant to attain a fuller picture of CSF dynamics especially regarding the improvement of VP shunt procedures. There were two sheep where minimal communication existed. The dimensions of the CSF space are considerably smaller than those of humans and it is possible that the implanted catheters caused a mechanical blockage in their respective spaces. The connection between the spinal and ventricular spaces may have indeed been present, albeit the communication may not have extended to the lateral ventricles.

## Conclusions

Our investigations have provided revealing insight into the intercompartmental relationships between ICP, ITP, ABP and CVP under physiological conditions. Volumetric pressure changes in the intrathecal space have been shown to propagate in both mean and pulse pressure to the intracranial and arterial systems consistently, and to a lesser extent to the venous system. Outflow resistance values did not correspond between calculation methods, further highlighting the need for an equation to unify constant pressure and bolus infusion methods.
